# Towards a Joint International Database: Alignment of SSR Marker Data for European Collections of Cherry Germplasm

**DOI:** 10.3390/plants10061243

**Published:** 2021-06-18

**Authors:** Matthew Ordidge, Suzanne Litthauer, Edward Venison, Marine Blouin-Delmas, Felicidad Fernandez-Fernandez, Monika Höfer, Christina Kägi, Markus Kellerhals, Annalisa Marchese, Stephanie Mariette, Hilde Nybom, Daniela Giovannini

**Affiliations:** 1 Department of Crop Science, School of Agriculture, Policy and Development, University of Reading, Reading RG6 6EU, UK; edward_venison@hotmail.co.uk; 2 NIAB EMR, New Road, East Malling, Kent ME19 6BJ, UK; Suzanne.Litthauer@niab.com (S.L.); felicidad.fernandez@niab.com (F.F.-F.); 3 INRAE-Unité Expérimentale Arboricole, Domaine de la Tour de Rance, 47320 Bourran, France; marine.delmas@inrae.fr; 4 Federal Research Centre for Cultivated Plants, Institute for Breeding Research on Fruit Crops, Julius Kühn Institute, Pillnitzer Platz 3a, 01326 Dresden, Germany; monika.hoefer@julius-kuehn.de; 5 Federal Office for Agriculture, Genetic Resources and Technologies, Schwarzenburgstrasse 165, 3003 Bern, Switzerland; christina.kaegi@blw.admin.ch; 6 Agroscope, Strategic Research Division Plant Breeding, Müller-Thurgau-Str. 29, 8820 Wädenswil, Switzerland; markus.kellerhals@agroscope.admin.ch; 7 Department of Agricultural, Food and Forest Sciences, University of Palermo, Viale delle Scienze-Ed. 4, 90128 Palermo, Italy; annalisa.marchese@unipa.it; 8 BIOGECO, INRAE, University of Bordeaux, Route d’Arcachon 69, 33612 Cestas, France; stephanie.mariette@inrae.fr; 9 Balsgård-Department of Plant Breeding, Swedish University of Agricultural Sciences, Fjälkestadsvägen 459, 29194 Kristianstad, Sweden; Hilde.Nybom@slu.se; 10 CREA-Research Centre for Olive, Fruit and Citrus Crops, via la Canapona 1 bis, 47121 Forlì, Italy; daniela.giovannini@crea.gov.it

**Keywords:** *Prunus avium*, sweet cherry, SSR, microsatellite, germplasm, genetic resource

## Abstract

The objective of our study was the alignment of microsatellite or simple sequence repeat (SSR) marker data across germplasm collections of cherry within Europe. Through the European Cooperative program for Plant Genetic Resources ECPGR, a number of European germplasm collections had previously been analysed using standard sets of SSR loci. However, until now these datasets remained unaligned. We used a combination of standard reference genotypes and ad-hoc selections to compile a central dataset representing as many alleles as possible from national datasets produced in France, Great Britain, Germany, Italy, Sweden and Switzerland. Through the comparison of alleles called in data from replicated samples we were able to create a series of alignment factors, supported across 448 different allele calls, that allowed us to align a dataset of 2241 SSR profiles from six countries. The proportion of allele comparisons that were either in agreement with the alignment factor or confounded by null alleles ranged from 67% to 100% and this was further improved by the inclusion of a series of allele-specific adjustments. The aligned dataset allowed us to identify groups of previously unknown matching accessions and to identify and resolve a number of errors in the prior datasets. The combined and aligned dataset represents a significant step forward in the co-ordinated management of field collections of cherry in Europe.

## 1. Introduction

Sweet cherry (*Prunus avium* L.) is grown widely around the temperate world as either a fruit or timber tree. The importance of conserving genetic diversity of agricultural crops is widely accepted and consequently, over 7000 accessions of sweet cherry are currently listed within the global Genesys database [1]; over 6500 are included within EURISCO, the European Search Catalogue for Plant Genetic Resources [2].

Providing access to data has been a priority of the European Cooperative program for Plant Genetic Resources (ECPGR) *Prunus* working group since its establishment [3] and, for example, standards for phenotyping have been established [4] in order to improve the consistency of characterization data. Sweet cherry is a challenging crop to distinguish at a morphological level and genetic markers offer significant value in efforts to compare between genebank holdings. The AEGIS (A European Genebank Integrated System) initiative [5] to develop a better co-ordinated European collection within ECPGR would benefit substantially from the ability to align and compare DNA marker data produced in different countries.

Microsatellite or simple sequence repeat (SSR) markers have been developed and tested for identification of cultivars in sweet cherry for a number of years [6,7,8,9]. In sweet cherry fruit trees they have been used to study genetic diversity in both local cultivars [10,11,12,13,14,15] and germplasm collections [16,17,18,19,20,21] and within groups of commercial cultivars [22], and to compare between domesticated cultivars and wild populations [23]. Following the ‘Ad hoc meeting on fingerprinting of *Prunus*, *Malus*/*Pyrus* and *Vitis*’ in 2006, a standard set of 16 SSR loci and eight reference genotypes were recommended for use in cherry within ECPGR [24]. These standards have been used in a number of studies [13,17] and the markers were able to discriminate known cultivars and confirm approximately 50% duplication within a range of material in dispersed Swiss collections [17] although, until now, no efforts have been made to align the data from different countries. More recently, the standards were reassessed during the ECPGR EU.CHERRY project [25] and an updated set of loci was recommended (Barreneche et al. personal communication). Within the EU.CHERRY project these loci were scored centrally across a range of representative accessions from a number of ECPGR genebanks in addition to a range of material supplied through the COST 1104 initiative.

SSR markers have remained the standard tool for germplasm management within ECPGR although, it has also been known for many years that data are prone to variability between laboratories [26]. Previously, efforts to align SSR data and create standardised databases have been made in tomato [27], wheat [28], maize [29], grape [30], cocoa [31], apple [32], olive [33] and currant/gooseberry [34] and, for example, salmonid fish [35,36]. Existing datasets have also been combined and complemented by the use of additional loci in order to carry out diversity analysis across groups of geographically representative collections in apple [37]. Most recently, and at least partially under the umbrella of ECPGR, significant efforts have been made to align a range of SSR data from a variety of sources in apple (Denance et al. personal communication). This approach has involved a wide range of collections, including both national germplasm collections and the collections of non-governmental organisations and has resulted in the allocation of a series of *Malus* UNiQue genotype codes (MUNQ) [38,39] (and Denance et al. personal communication).

The fine detail of these approaches has been subtly different, to an extent dictated by the circumstances of each individual study. For instance, both standardised [29] and non-standard [30,33,34] genotyping protocols have been used with similar success. Common to all of them is a use of references in order to standardize allele calling. The type of reference used has ranged from the level of the cultivar [32] to the level of the single amplicon [31]. The majority of approaches tend to identify the use of either reference genotypes (for instance at the accession level in a single genebank), or reference samples (for example, post DNA extraction) as a reasonable and pragmatic solution to the need to effectively replicate standard alleles across laboratories. The consistent aim of all these approaches has been to reproduce as complete and wide a range of the expected alleles as possible.

Here, we present a first attempt to align SSR data from a number of significant European germplasm collections of sweet cherry. We bring together the previous work of national collections in genotyping their own germplasm using standard marker sets (national datasets) with the international (and central) dataset produced in the EU.CHERRY project. Where national datasets contain representatives of sour (*Prunus cerasus* L.) and hybrid ‘Duke’ (*Prunus* × *gondouini* (Poit. and Turp.) Rehder) cherry we included these. We supplement the existing central data with additional genotyping, in order to expand the coverage of allele scores for comparison between datasets, in an attempt to begin to produce an aligned dataset for comparison of sweet cherry germplasm holdings within Europe.

## 2. Results

### 2.1. Standardisation of the Great Britain (GB) Dataset

In 41 out of 53 samples theoretically replicated between the two phases of GB data, a consistent alignment factor could be identified (Supplementary Table S1). The remaining twelve samples were deemed to represent either collecting or data handling errors in one or other analysis. Three of the twelve errors appeared to reveal profiles, which agreed with either the locus- or allele-range-specific alignment factors in only one multiplex, suggesting a data handling error, and the remaining nine could be identified to align consistently to different samples in the dataset, suggesting a sample handling error. Thirty-two further samples from the first phase of analysis, which had been deemed to represent duplicates in the collection to those used for comparison, were in general agreement with the locus- or allele-range-specific alignment factors. For the locus EMPaS02, an allele-range-specific alignment factor was deemed necessary, with alleles of 146 bp and 148 bp being adjusted by 4 bp and the remainder of alleles adjusted by 3 bp based on the majority of examples. For all but one allele call (a difference of 15 bp called in EMPaS12), the size of an error in alleles that did not align by the locus- or allele-range-specific factor was within ± 1 bp of the factor. Within the set of 41 replicated samples, the percentage of allele calls that aligned by either the locus- or allele-range-specific alignment factor ranged from 77% to 99% with an additional 1–16% represented by either missing data or null alleles (Table 1).

### 2.2. Production of a Central Dataset

In total, 191 alleles were scored across the 99 samples and ECPGR reference genotypes used to generate the central dataset for alignment. Allele number per locus ranged from 7 (CPSCT038) to 20 (BPPCT034). By comparison to the already available EU.CHERRY dataset the new data contained an additional 58 alleles (including 19 that were only produced in the ECPGR reference genotypes) and thus expanded the range for comparison. In a series of 22 samples that aimed to replicate data from the EU.CHERRY analysis (including twelve of the German samples deemed to have originally been mislabelled in the EU.CHERRY dataset, following realignment using their inferred profile name) the data replicated here were in almost complete agreement with the EU.CHERRY data; the main exception being a consistent 2 bp shift in scores between the EU.CHERRY data and the data generated here for locus EMPa017 (Supplementary Table S2). Compensation for this was allowed in all further comparisons to the original EU.CHERRY dataset and a 2 bp adjustment was made to the EU.CHERRY data for this marker in the aligned dataset (Supplementary Table S3).

In many cases, low signal quality data was obtained from loci EMPa017 and CPSCT038. No alleles were observed from locus CPSCT038 for the reference genotypes *P*. *incisa* E621, or *P*. *nipponica* F1292 and sample SLU005 (‘Pernilla’, Sweden), despite repeated attempts at PCR amplification. In addition, the large allele sizes observed from locus PAV-Rf-SSR made it necessary for this locus to be scored separately from other loci in the software.

### 2.3. Alignment of National Datasets to the Central Data

Locus- or allele-range-specific alignment factors could be identified from 173 out of the 196 profiles used for comparison between the national datasets, including ECPGR reference genotypes where used, and the central data (Supplementary Table S4). The remaining 23 profiles (representing 17 accessions, six of which were sampled twice) were deemed to have been caused by collecting errors in either the national dataset or the data replicated here for alignment. In all six repeated analyses, data were consistent between replicates in our analysis and the previous analysis in EU.CHERRY and therefore indicated an error in the national dataset. One additional sample (V2799 Adelise^®^ Masdel in the French dataset) was previously noted to be a likely error in the national data.

Within the set of 173 samples used to generate the locus- or allele-range-specific alignment factors, the minimum percentage of allele calls that aligned by the factor was 46% and the percentage in the remaining comparisons on a country-by-country basis ranged from 60% to 100% (Table 2). Up to 52% of additional comparisons were represented by either missing data or null alleles and together, between 67% and 100% of comparisons were either in agreement or appeared to either represent or align to null alleles. Only seven allelic comparisons differed from the locus- or allele-range-specific alignment factor by what was considered to be a large amount (ranging from 8 to 28 bp away from the factor) and these were deemed to be likely calling errors in one or other dataset.

Overall, 2873 allelic comparisons were made across 480 different alleles called within the national datasets and ECPGR reference genotypes. Of these, a locus- or allele-range-specific alignment factor was supported across 376 alleles (Supplementary Table S5) based on the 2481 allelic comparisons that were deemed to be in agreement. A further 211 individual allelic comparisons were deemed to be confounded by null alleles in one or other dataset and 181 individual allelic comparisons were judged to be in disagreement with either the locus- or allele-range-specific alignment factor.

For 104 of the alleles that were compared it was not possible to find a majority of comparisons in agreement with the locus- or allele-range-specific alignment factor. Seventy-one of these were incorporated as allele-specific adjustments in the final alignment factors on the basis that the comparisons for the alleles in question disagreed with the locus- or allele-range-specific factor by a consistent amount that was conceivably caused by either differential calling or rounding of scores (Supplementary Table S5). In 33 of the 71 cases the allelic comparisons in disagreement represented all of the entries for the allele call in the national dataset (the majority only being represented once). Three further cases were accepted on the basis that one (the adjustment of a single entry scored at 105 bp for EMPa002 in the Swedish dataset) was uniquely associated with the single entry at 103 bp for the same locus and both were in disagreement with the locus-specific alignment factor (i.e., the exception was based on a unique sample), and two were identified as known exceptions in the 2018 entries of the Swiss dataset based on local knowledge (but were not included for reproduction in our analysis).

Twenty-four unresolved queries were noted to remain in the data alignment. In a number of these, the final alignment factor based on the central dataset appeared to be in potential conflict with the alignment between national datasets. For example, by comparison to the central data, the alleles originally called at 120 bp and 126 bp for locus BPPCT037 in both the Italian and Swedish data were all adjusted by 1 bp on the basis of disagreement with the locus-specific alignment factors despite the factor in both datasets being to retain data as it was (i.e., the datasets were in closer agreement before the application of the allele-specific adjustment in the final alignment factor than they were afterwards). Twenty-one out of 24 of the unresolved queries involved allele calls made in the ECPGR reference genotypes.

Fifty-two final alignment factors were generated and in 33 of them the alignment factor was judged to be largely consistent throughout the allele range, i.e., was based on a locus-specific alignment factor (Supplementary Table S6). In 13 of those based on allele-range-specific alignment factors the alignment factor was judged to be variable across the allele range (in many cases with a clear size-related shift). In the seven remaining cases based on allele-range-specific alignment factors, the alignment factor was judged to be dependent on whether alleles were scored as odd or even (including one where there was also a size-related shift). Of these 52 final alignment factors, 31 were improved through the inclusion of allele-specific adjustments.

Following the adjustment of data, ten of the examples where profiles were deemed to be collecting errors during comparison could be aligned with alternative samples, largely in the same national dataset. One further example (1987-128 Ferbolous in the GB data) appeared to consist of a chimeric profile that was correct for MP1 but identifiable to a different sample for MP2. In one particularly interesting example, the profile generated here for the purpose of alignment from accession Pa_248 Wils Frühe from the German collection agreed completely with the sample in the GB national dataset for 2002-106 Wils Fruhe Herz following data alignment. This finding suggests that the Pa_248 SSR profile in the German dataset is in error, but that the accession in the German collection is correct and in agreement with its GB counterpart as part of the same group (Supplementary Table S3).

### 2.4. Diversity Metrics for the Aligned Dataset

Allele number per loci in the subset of 1302 unique diploid entries ranged from six (CPSCT038) to 22 (CPPCT022 and BPPCT037) with a mean of 14.5 (Table 3). Observed and expected heterozygosity ranged from 0.35 and 0.37 in EMPa017 to 0.83 and 0.84 in EMPaS06 respectively with a mean value of 0.67 for both measures. One-hundred and nine additional alleles were included in the non-diploid entries giving a total of 312 alleles reported across the 14 loci (Table 4).

### 2.5. Identification of Matching Accessions

In total, 63 groups of matching accessions were identified (Supplementary Table S3). The largest group (group 3) contained 56 members and included three samples that were scored in our analysis (V1929 Belge from France, 1968-129 Magyar Porc Csereszyne (HTB) from GB, and Pa_5 Schneider’s Späte Knorpel (kirsche) from Germany) and the ECPGR reference genotype ‘Noir de Meched’. In addition to the GB and German samples (which were included in both analyses) a further eleven members were analysed in EU.CHERRY and included representatives from Bosnia and Herzegovina, Italy, Hungary, Slovakia, Morocco and Austria, all held under different accession names. The remaining members were contained in the national datasets and included the three entries for the ECPGR reference genotype used in Sweden, Italy and GB and national data for the French, German and Italian accessions analysed either here or in EU.CHERRY along with 33 further examples. Some of the additional samples were known to be indistinguishable and nine (one German and eight Swiss samples) were labelled with names associating with ‘Schneider’s Späte Knorpel’, a further four (one French, three Swiss) were labelled with versions of ‘Badacsony’; two of the British samples were noted to be likely handling errors and it is possible that at least one of these had been mixed up with the British accession that was analysed both here and in EU.CHERRY (1968-129 Magyar Porc Csereszyne (HTB)) because the entry for the accession in the national data was noted to be in error and identified within a different group. At least twelve further names were listed for members of the group, although at least one of these (a sample labelled Napoleon in Switzerland) was clearly in error.

The next largest group (group 23) contained 42 members including one sample that was scored in our analysis (PA_2 Hedelfinger from Germany). This accession was also analysed in EU.CHERRY (although had been mislabelled in the EU.CHERRY dataset according to our findings) along with six other representatives from the Czech Republic, Bosnia and Herzegovina, Slovakia and Belgium, again all held under different accession names. Again, the remaining members were held in the national datasets and included national data for the German accession analysed in both EU.CHERRY and our analysis. Of the remaining 33 members the vast majority (30) were Swiss with one further member from France and two from GB although one of these (an entry labelled Kordia in the British data) appeared a likely labelling error because by name it should have been a member of a different group. Within these samples, twelve accessions were associated with the name Hedelfinger although at least 17 further names were listed for members of the group, at least one of which (the entry labelled Kordia as above) was most likely in error. Two of the Swiss accessions (represented by three data entries) had been renamed Hedelfinger following initial analysis.

Eight further groups (groups 2, 4, 6, 9, 14, 25, 30 and 44) contained more than ten members. Seven of these included groups that appeared to associate around the cultivar names: ‘Napoléon’; ‘Noble’; ‘Dönissens Gelbe Knorpelkirsche’; ‘Bigareau Moreau’/’Souvenir de Charmes’; ‘Early Rivers’/’Kaštánka’; ‘Büttners Rote Knorpelkirsche’ and ‘Basler Adler’. With the exception of the latter group (which was comprised of twelve entries from Switzerland and one from Germany) all of these groups contained representatives from between three and five of the partner countries in our analysis and between five and eight of the countries represented in the EU.CHERRY dataset. The only other group with more than ten members included eight different names and none of these was repeated between countries (accession names in Germany and France included Drogans Gelbe Knorpel (kirsche) and Grosse blanche de Verchocq respectively). Additional representatives of this group from four further countries were included in the EU.CHERRY dataset.

## 3. Discussion

Our findings demonstrated that we were able to produce a reasonably well aligned dataset encompassing data scored on at least nine different occasions across six different countries through alignment against two sets of centrally scored replicates. The aligned dataset contains 2241 entries for national accessions in: France (206 entries), GB (406 entries) Germany (362 entries), Italy (193 entries), Sweden (52 entries) and Switzerland (1023 entries) alongside the 99 samples and eight ECPGR reference genotypes analysed centrally. The dataset also contains the 324 EU.CHERRY profiles aligned to the data we present here (Supplementary Table S3).

Through the establishment of a series of locus- or allele-range-specific alignment factors, based on the majority of allelic comparisons in our theoretically replicated samples, it was possible to identify and exclude data that were caused by sampling or handling errors; in many cases it was possible to identify the erroneous sample subsequent to applying the final alignment factor. The aligned dataset allowed us to identify a range of accessions, which, using the markers we report, are genetically indistinguishable between national collections. Some of these might have been expected based on accession name, and others have been previously reported [13,17,19] but many remained unknown until now.

### 3.1. Consistency of Alignment Factors

In the absence of either collecting or handling error, the alignment factors were generally consistent between samples with the majority of allele comparisons being in agreement with either a locus- or allele-range-specific factor (ranging from 74% of comparisons in the GB internal data alignment and from 46% in the alignment of national datasets). Acknowledging instances of null alleles allowed for more than 77% and 67% of comparisons respectively to be accounted for. In the example with the lowest percentage agreement (46% for locus Pav-Rf-SSR in the GB data) an additional 52% of comparisons were marked as null alleles, suggesting a likely problem with either amplification of the locus or scoring of the marker rather than an inconsistency in the scored data.

Given that the two phases of GB data were both analysed on the same system, it would be expected that the data might align more closely and more accurately and this was generally the case with the majority of alleles requiring no adjustment. The exceptions to this were BPPCT037 that required a 1 bp adjustment, EMPaS02 that required either a -3 or -4 bp adjustment and EMPaS12 that required adjustment by -8 bp. The only known changes between the two phases of analysis were that the dye was swapped from 6-Fam to Pet on the primer for EMPaS02 and from Ned to 6-Fam on the primer for EMPaS12, although on checking it was also noted that the forward primer for EMPaS12 was missing the last three bases at the 3′ end (due to an error at the time of ordering). The potential for the fluorescent label to affect capillary electrophoresis is accepted [40] although, in a detailed study and review of ‘dye-shift’ as a source of genotyping error [41] the authors reported that, whilst shifts due to changes in dye could cause errors in the range of 2.07–3.68 bp, there was a tendency for Pet labelled fragments to be scored the largest and for 6-Fam labelled fragments to be scored as the smallest (with Ned and Vic being close to, or potentially slightly larger than 6-Fam). The difference in size of our allele calls is inconsistent with this finding and it would appear that ‘dye-shift’ might not explain the differences. Equally, given that the shortened primer was evidently still capable of producing a consistently scoreable fragment, there would be no logical reason why a reduction at the 3′ end would result in any change in fragment size. Furthermore, the second phase (and adjusted) data were scored more closely to the central dataset (which used the full-length primer, although this was labelled with Hex). Given that the remaining loci, which were analysed on the same equipment, required no internal adjustment in the GB data, we are unable to explain this finding but nonetheless, the difference between phases was consistent.

The size of alignment factor required between the Swiss samples and the central dataset was to be expected and can at least in part be explained by the use of 5′ tailed primers in the generation of the Swiss data. This was noted previously where an average difference of 9.4 ± 1.5 bp was reported in comparison to the published scores for the ECPGR reference genotypes [17]. The majority of locus or allele-range-specific alignment factors for the Swiss data were close to this size range. The differential scoring specific to marker CPPCT022 in the Swiss data, whereby a number of alleles scored in samples post 2018 were consistently reported at 2–3 bp larger (resulting in a number of allele-specific adjustments to the final alignment factor) is not fully explainable; the issue was associated with a change in the analytical laboratory and appears to be related to fragment sizing against the electrophoresis size standard. The size difference had already been identified locally, and we incorporated this local knowledge within our alignment. Similarly, a reasonably consistent, and as yet unexplained difference was seen in the scoring of EMPa017 between the EU.CHERRY analysis and our dataset. Again, this was factored within the alignment and subsequent comparisons.

### 3.2. Selection of Reference Samples

It is clear that the key to aligning datasets produced in different laboratories is the inclusion of a series of reference samples. In our analysis, we used a combination of the recommended ECPGR reference genotypes and a set of ad-hoc selections (based on the datasets of each collection) for alignment. Through the addition of these collection-specific samples we were able to expand the number of alleles represented from 102 in the ECPGR reference genotypes to 172 in our central dataset. Of the outstanding queries that we were unable to resolve, it is notable that the vast majority (21/24) arose from alleles called in the ECPGR reference genotypes and this might be taken to suggest that a number of the currently recommended reference genotypes, or alleles within them, are difficult to score. It is also notable that 32 out of 71 allele-specific adjustments that were made to the final alignment factors were based only on scores from the ECPGR reference genotypes and these would not have been possible to make without the ECPGR references being included. However, 18 of the allele-specific adjustments based on the ECPGR reference genotypes were in alleles unique to the reference genotypes themselves, and this potentially both explains the difficulty in their calling and brings into question their value for data alignment. The ECPGR reference genotypes were originally selected with the view of representing as wide a range of diversity as might be expected [24] and, whilst our study did place a focus on sweet cherry, this finding could also be taken as an indication that our collections would benefit from wider diversity. Nonetheless, it would potentially be worthwhile to reconsider an amended set of reference genotypes that would align better with the range of alleles that are commonly found in sweet cherry collections in the future. It is also notable that in none of the datasets we present (including the central dataset on which our alignment is based) were the allele scores aligned to the actual published allele sizes [24] for the reference genotypes, and any attempts to align with data that have been adjusted to match the published scores will require a further stage of realignment to our dataset.

It is likely that further improvements could be made to our alignment in the future if additional samples were replicated in order to resolve the outstanding queries. Ultimately, allelic size standards such as those developed for Cocoa [31] could allow the further improvement, and similar approaches have been used for a long time in humans [42,43]. In addition to SSR markers, single nucleotide polymorphism (SNP) markers for use in cherry have been available for a number of years [44,45] and these have been proposed as a potentially more rapid and cost-efficient marker for cultivar identification [44,46]. Any change in technology would require the existing dataset to be reproduced and new methods of standardization to be considered and, realistically, both of these remain beyond the scope of any current activity within ECPGR.

### 3.3. Identification of Errors Through Data Alignment

Perhaps unsurprisingly, the datasets used for alignment contained a range of errors. Most of the data had not been replicated (as is often the case in genebank management, often due to limitations in funding) and until attempts were made at alignment a number of these errors were not detectable. In total, 97 profiles are noted to represent potential errors within the national datasets and the data we produced for alignment, a further seven erroneous profiles (the incorrectly ordered ECPGR reference genotypes in the GB data) are marked as resolved through the acceptance of inferred profile names, resulting in an error rate of approximately 4%. In addition to this we identified two outstanding queries and 18 errors (resolved through the use of inferred profile names) in the EU.CHERRY dataset. In cases where the reason for disagreement could not be resolved, both entries were marked as potential errors and, whilst we would expect only half of these to actually be in error, it is also likely that there will be further errors that can only be identified through the consideration of matching accessions that are supposed to represent genetically different cultivars. On further analysis of the profile for the German sample of Wils Frühe in the central dataset, it was apparent that it is an exact match to the entry for accession Pa_205 Kunzes Kirsche in the German national dataset and the simplest explanation for this would be that a handling error was made in the generation of the national data. Handling errors between genetically unique accessions will remain undetectable until data are reproduced.

It was notable that, in addition to whole sample handling or labelling errors, the replication and alignment of data was able to help identify errors within profiles, including where handling errors appeared to have been made between the different multiplexes, resulting in the creation of a chimeric profile. The majority of allele-specific adjustments that were included in the final alignment factors were scores within 1–2 bp of either the locus- or alle-range-specific alignment factors and all of those that were accepted were conceivably caused by either rounding or calling error. Any profiles that differ more significantly from either the locus- or allele-range-specific alignment factors, and especially those that differ inconsistently between loci should be treated with caution. We also noted examples where the allele-specific adjustments, based on alignment to the central dataset resulted in allele sizes from national datasets becoming less close than they had been prior to alignment. It is possible that this indicates an inconsistent, or incorrect call in the central data and any analysis of the aligned dataset should allow for the fact that it is possible that such instances have been applied in our alignment.

We were able to correct, what appeared to potentially have been a systematic error in the EU.CHERRY analysis, in that a set of the German samples had been realigned during either DNA extraction, analysis or data handling. It was not possible to trace where the error had occurred, but it was reassuring, and would seem to support the accuracy of our alignment that we were able to match all of the samples to their correct genotype, with the exception of one that appears to be in error in the German dataset.

### 3.4. Comparison to Alignment Attempts in Other Species

The challenges of aligning genetic fingerprint data have been known for many years and the equivalent issues that that had been experienced in the salmonid fish research community, beginning with attempts to align allozyme data, were reviewed by Moran [47]. Further researchers working on maize lines have reported finding 45% of errors between datasets to be correctable frameshift errors, with an additional 16% being attributed to ‘null’ alleles [29]. Others have reported that the majority of errors were “easily identifiable rounding errors” [33]. The range of lab-to-lab variability for alleles of a single locus has been reported as 0–7 bp [34] with the maximum variability in the cited examples being 17 bp [36] although, in the study in question only one out of 14 loci had a maximum difference greater than 13 bp. It would appear that the differences we have encountered in our data alignment are comparable to these previous findings.

### 3.5. Discriminatory Power of the Aligned Dataset

The discriminatory power of individual SSR loci is known to vary, and authors have previously reported that all accessions in their studies have been able to be distinguished by as few as five [22] or six [11] loci. A subset of four of the originally recommended ECPGR loci [24] was found to be sufficient to discriminate between the majority of a relatively small set of Ukrainian cultivars [15] however, two of these loci (PceGA34 and UDP98-412) had been excluded from the recommended set of loci in the EU.CHERRY project and consequently these were not included in our alignment. The ability to discriminate will be influenced by the number of loci that are scored in each dataset. The lowest probability of identity (PID) that was calculated for any entries considered as matching in our analysis was 7.47 × 10^−5^ (when assuming that samples were unrelated) or 1.75 × 10^−2^ (when assuming that samples were full sibs) although this was for the identification of the Swedish entry generated from the ECPGR reference genotype ‘Noir de Meched’ as a member of group 3, along with the other representatives of the reference genotype. The next lowest PID value for an entry that was considered as matching was 2.73 × 10^−5^ (when assuming that samples were unrelated) or 1.72 × 10^−2^ (when assuming that samples were full sibs) for the identification of the Swedish accession of Allmän Gulröd Bigarrå in Brunstorp as a member of group 27 along with the GB accession 2002-160 Kent Bigarreau, three French accessions (held under the names Big de Fontainebleau, Gros bigarreau rouge et jaune and Big Hâtive productif) and the German accession Pa_196 held under the name Weiße Spanisch and a Norwegian accession in the EU.CHERRY dataset under the name Kvit Spansk (translated to “White Spanish” in English); the remaining members of the group were identified with larger numbers of matches and much lower PID values on account of having more data for comparison. A second Swedish accession under the same name at the Elite Plant Station appeared to be clearly different, as did a third accession under the same name in the GB collection, although this latter accession was identified to match accessions of ‘Hudson’ in both Germany and Switzerland and may well represent an error in either the GB collection or dataset). Clearly, a number of these groupings will require further resolution. It is also likely that there will be additional groups of matching accessions that we have failed to identify, and that some of those that we identify here may be brought into question by additional genotyping or morphological analysis in the future.

### 3.6. Genetic Diversity in the Aligned Dataset

The aligned national data in the final dataset contained a larger number of alleles than were reported in the analysis of national datasets [13,17,23]. Within the diploid samples, a higher allele number was found for eight out of the 14 loci when comparing the aligned national data with the central dataset (including the EU.CHERRY data); the remaining loci were (with the exception of EMPa017) only scored in a maximum of two national datasets (and scoring for PAV-Rf-SSR in the GB dataset was partial). It would appear that the increase in allele number is at least partly linked to the increase in sample size, although it was also increased further in the overall dataset through the inclusion of hybrid and sour cherry genotypes, which are known to contain additional alleles for some loci [17]. Any increase in allele number that cannot be accounted for might be investigated further to confirm whether it could be an artefact of either inconsistent scoring or alignment. The values of expected and observed heterozygosity in the diploid samples from the aligned national data were, again, similar to those reported in the analysis of national datasets prior to alignment [13,23] and it would appear that this value has not been affected significantly in the aligned dataset. We have not attempted to analyse genetic structure further, because only a relatively small number of loci were scored across all datasets. A more detailed study using the full set of loci is being carried out on the EU.CHERRY dataset (ref the INRAE study).

### 3.7. Summary

The primary objective of our study was to allow the comparison of holdings of cherry germplasm across a series of major European collections. In doing that we have been able to identify a range of groups of accessions that, at least using the markers we present, are genetically indistinguishable. Many of these revolve around well-known and historic cultivars that are likely to have been well-distributed and are consequently likely to have both become renamed and misidentified in situ. It is not possible at this point to resolve the issues of synonymy versus mislabelling, and it is certain that a number of the indistinguishable groupings will contain sports and clones that have value in themselves. A more detailed consideration of the indistinguishable groups remains necessary to fully resolve these matches. However, the aligned dataset that we have produced represents a significant step forward in the co-ordinated efforts to conserve germplasm of cherry within Europe.

## 4. Materials and Methods

### 4.1. National De Novo Genotyping of GB Samples

Genotyping of GB national samples was carried out in two phases. Initially, DNA from 92 samples suspected to represent duplicates in the collection was extracted from fresh leaves using the QIAGEN DNeasy 96 Plant Kit (QIAGEN, MD, USA) according to the manufacturer’s protocol. Amplification was carried out in 11 µL volumes using the Type-it™ Microsatellite PCR kit (QIAGEN, MD, USA) according to the manufacturer’s protocol, with 0.2 µM of each primer allocated to four previously described multiplexes [17], which were modified by the addition of the labelled primers 6-Fam-EMPa002 to multiplex C6, 6-Fam-EMPa017 to multiplex C7 and 6-Fam-EMPa003 to multiplex C8. Thermal cycling was carried out in a Veriti 96 well thermal cycler (Applied Biosystems, MA, USA) as follows: initial 5 min denaturation at 95 °C; 28 cycles comprising a 30 s denaturation step at 95 °C, followed by 90 s of annealing at 55 °C and 30 s of extension at 72 °C and a final extension step of 30 min at 60 °C. Secondly, DNA from 312 samples representing the remainder of the collection, and theoretically replicating 53 of those analysed previously was again extracted from fresh leaves as above. Primer combinations for multiplex were: Multiplex A (including the labelled primers 6-Fam-EMPa002, Vic-CPPCT022, Vic-CPPCT006, Pet-EMPaS02 [24]; 6-Fam-CPSCT038, Pet-BPPCT034 [48]; Pet-PAV-Rf-SSR [49]) and Multiplex B (including the labelled primers Ned-BPPCT037, Ned-EMPaS06, Vic-EMPa004, 6-Fam-EMPa017, Ned-EMPa018, 6-Fam-EMPaS12 and Pet-EMPaS14 [24]). In later and any repeated analysis Multiplex A was split such that EMPa002, CPPCT022 and CPPCT006 were amplified and separated together whilst CPSCT038 was amplified independently and added to a second multiplex containing BPPCT034, PAV-Rf-SSR and EMPaSO2 for the purposes of separation as this appeared to make amplification more consistent and allele calling more straightforward. Thermal cycling was carried out in a Veriti 96 well thermal cycler (Applied Biosystems, MA, USA) as follows: initial 5 min denaturation at 95 °C; 10 touchdown cycles comprising a 30 s denaturation step at 95 °C, followed by 90 s of annealing starting at 55 °C in the first cycle and decreasing 0.5 °C per cycle, and 30 s of extension at 72 °C; 20 cycles of 30 s at 95 °C, 90 s at 50 °C and 30 s at 72 °C; and a final extension step of 30 min at 60 °C. Following amplification, PCR products obtained for both phases of the GB investigation were separated on an Applied Biosystems 3730xl capillary sequencer by Source Bioscience (Nottingham, UK) using a LIZ 500 standard. Alleles were called in GENOTYPER software (Applied Biosystems, MA, USA) and a consensus score was created for all samples where analysis was repeated using the split multiplex.

### 4.2. Compilation of National Datasets and Central Data from EU.CHERRY

SSR data from the remaining partner countries were produced and scored as described previously: France [23], Germany [21,50,51], Italy [13], Sweden [19], Switzerland [17,20] and datasets from these analyses were supplied by the partners for compilation. Centrally produced SSR data from the EU.CHERRY project were also made available.

### 4.3. Selection of Additional Reference Accessions

Each national dataset was initially screened in order to identify the range of alleles that were scored for each locus. Alleles that could already be aligned through samples that were replicated in the EU.CHERRY dataset were marked, along with alleles that were attributed to the ECPGR reference genotypes where these were included. Additionally, alleles that were only reported in polyploid profiles were marked, on the basis that these were potentially specific to accessions of *Prunus cerasus* L. or hybrids and therefore of less use for the main alignment.

A series of accessions that would be expected to replicate as many as possible of the remaining alleles in each dataset was then selected following an empirical approach. Specific emphasis was placed on trying to ensure that alleles were replicated from the limits and being distributed across each range. The list of selected accessions was further modified on the basis of the availability of material and/or ease of collection if samples were geographically distributed and resulted in the following numbers of accessions being identified for replicated analysis: France (18); Germany (31) Italy (12); Sweden (8); Switzerland (7); GB (23 accessions). Fresh leaf samples from all 99 accessions were supplied to NIAB-EMR by the various partners. Leaf discs were preserved on silica gel on arrival.

### 4.4. Expansion of the Central SSR Dataset

DNA extractions were performed on all 99 samples at NIAB-EMR using the protocol described by Edge-Garza et al. [52]. Polyvinylpyrrolidone (PVP) was used and 6 M ammonium acetate was replaced with 5 M sodium chloride. DNA pellets were resuspended in 10 mM Tris-HCl pH 8.0 and diluted to 5 ng/µL for use.

PCR reactions were carried out using two multiplexes: Multiplex A (including the labelled primers 6-Fam-EMPa002, 6-Fam-CPPCT022, Hex-CPPCT006, Pet-EMPaS02 [24]; 6-Fam-CPSCT038, Ned-BPPCT034 [48] and Hex-PAV-Rf-SSR [49]) and Multiplex B (including the labelled primers 6-Fam-BPPCT037, 6-Fam-EMPaS06, Pet-EMPa004, Pet-EMPa017, Pet-EMPa018, Hex-EMPaS12 and Hex-EMPaS14 [24]). Amplification was performed in 13 µL volumes using the Type-it™ Microsatellite PCR kit (QIAGEN, MD, USA) according to the manufacturer’s protocol, using 0.2 µM of each primer, and a Veriti 96 well thermal cycler (Applied Biosystems, MA, USA) following the same touchdown program as the later analysis of GB accessions (above). The eight ECPGR reference genotypes: F12/1; ‘Goodnestone Black’; ‘Napoleon’; ‘Noble’; ‘Noir de Meched’ (all *P. avium*), *P. incisa* E621, *P. mahaleb* SL64 and *P. nipponica* F1292 [24] were included in each plate such that at least one standard was included per 16-capillary injection.

Following PCR amplification, products were diluted (1:10) and 1.3 µL separated using an ABI 3130xl Genetic Analyser (Applied Biosystems, MA, USA). Data were collected and alleles sized using GENESCAN and GENOTYPER software applications (Applied Biosystems, MA, USA). Estimated allele sizes were rounded and harmonized using Excel (MS Office) following automated scoring and sizing in GENOTYPER software (Applied Biosystems, MA, USA). Allele sizing was harmonised across plates according to the profiles obtained for the ECPGR reference genotypes. Data were compiled alongside profiles generated in the EU.CHERRY project following the same protocol. All additional central data we present are labelled ‘*Prunus* Alignment data’ in supplementary tables.

### 4.5. Alignment of Data and Estimation of Alignment Factors

The two phases of GB data were aligned by comparing allele scores across the 53 theoretically replicated samples. Locus-specific alignment factors, based on the majority of scores across all replicated alleles, were initially calculated. Where specific groups of alleles (e.g., within a specific size range) stood out as aligning differently to the majority, an allele-range-specific alignment factor was calculated as an improvement on the more general locus-specific factor. These alignment factors were applied across all of the first phase data in order to generate a single, consistently scored GB dataset. In four cases, the comparison was based upon alignment to a different accession that was known to be indistinguishable, rather than a technical replicate, because the sample for the replicate in the second phase data was deemed to represent a collecting error (Supplementary Table S1). Comparisons that differed from the locus- or allele-range-specific alignment factors across multiple loci were excluded on the basis that they were deemed to represent errors in one or other dataset.

Data supplied by the partner countries were aligned by comparing allele scores across samples that were reproduced within the central dataset (including the previously generated EU.CHERRY project data) in addition to the ECPGR reference genotypes where these were used. In total, 196 profiles were aligned, representing 146 national accessions (24 of which were sampled in both EU.CHERRY and our analysis and seven of which were national representatives of the ECPGR reference genotypes). Again, a locus-specific alignment factor was initially calculated based on the majority of scores, and where specific groups of alleles (including both size ranges and, e.g., groups of odd or even scores) stood out as aligning differently, this was improved to form an allele-range-specific alignment factor. In a number of instances (primarily relating to a set of 18 German samples analysed in EU.CHERRY) it was clear that the EU.CHERRY data were in error and in the case of the German samples it was found it was possible to reorder the profiles such that they agreed with the original delivery protocol, rather than being in alphabetical order (as was the case in the EU.CHERRY dataset). This rearrangement brought all but one profile in line with the locus- and allele-range-specific alignment factors for the German dataset and so the reordered profile names were inferred to be the correct profile names. A similar situation was identified in the GB national dataset where the ECPGR reference genotypes were evidently out of order and again, the correct profile name was inferred on the basis of reordering these profiles. In one additional case, an EU.CHERRY profile under the name Sämling aus Sauerbrunn was compared to the sample Ironsides in the knowledge that this was a labelling error in the EU.CHERRY dataset. Again, following the acceptance of these ‘inferred profile names’, any comparisons that differed from either the locus- or allele-range-specific alignment factors across multiple loci were excluded on the basis that they were deemed to represent errors in one or other dataset (Table S4).

Both locus- and allele-range-specific alignment factors were subsequently improved when a difference from either factor was indicated by the majority of cases used for comparison of any individual allele and where the difference in that allele was judged not to deviate greatly from the locus- or allele-range-specific factor. Thus, through an iterative process, a ‘final alignment factor’ was generated and this was either based upon a single locus-specific adjustment, a set of adjustments specific to ranges of alleles, or an improved version of either of these including further allele-specific adjustments as appropriate. This ’final alignment factor’ was then applied across the range of alleles in each national dataset.

### 4.6. Identification of Matching Accessions

**Figure 1:**
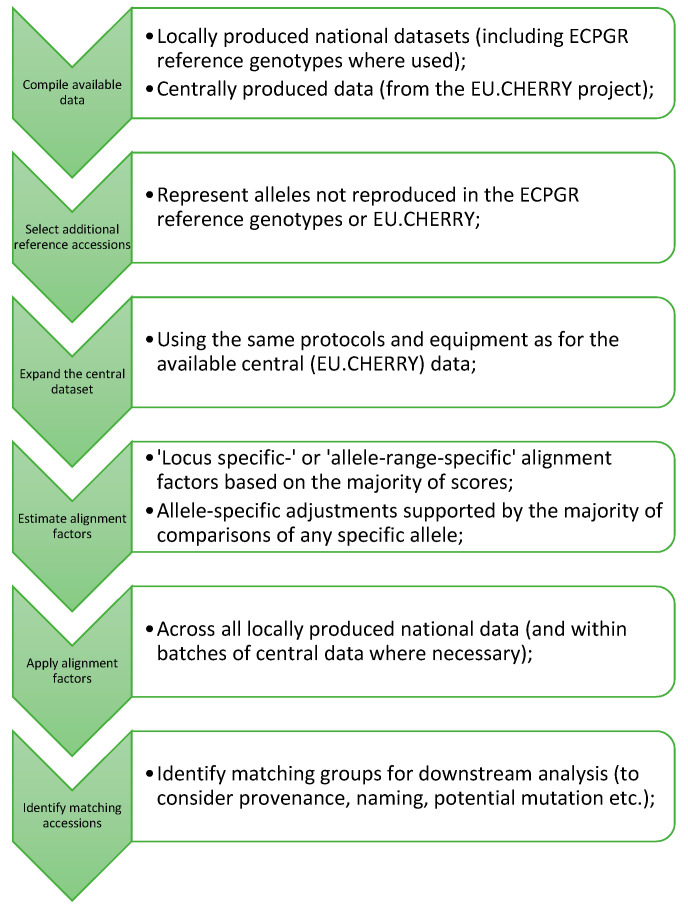
Summarised process for the alignment of datasets from national collections. Generic stages in the alignment process are listed in arrows with detail specific to our datasets and process expanded in the accompanying bullets.

Matching accessions were identified using the software Cervus 3.07 (Field Genetics, London, UK). Prior to analysis, a ‘false diploid-only’ dataset was created such that only the first two alleles reported for each marker (in increasing size order) were used for comparison of triploid and tetraploid profiles. A relatively low basic level of identity (requiring a minimum of only four matches and allowing one mismatch) was used to identify potential matches within Cervus. These matches were subsequently screened in Excel (MS Office) to allow a differential level of identity to be applied to matches within each national dataset in accordance with the differing numbers of markers for which data were available. Consequently, the minimum number of matches accepted was set to: 5 (Sweden), 6 (Italy), 7 (France/Switzerland), 8 (Germany) and 10 (GB). Comparisons were generally limited to matches between different national datasets and between national datasets and the central data. Following the identification of groups, samples with the same name that were only excluded due to missing data were also included as group members. A suggested process for the alignment of similar datasets is summarised in Figure 1.

### 4.7. Generation of Diversity Metrics

Genetic diversity metrics (allele no., observed and expected heterozygosity, polymorphic information content and average non-exclusion probabilities) were calculated in Cervus 3.07 (Field Genetics, London, UK). Prior to analysis, a diploid-only dataset was extracted from the aligned data by excluding any entries containing three or more alleles for any locus. The data entries were filtered to remove duplicate entries based on all data (rather than as identified as matching accessions above) and the remaining unique diploid entries were used to generate allele frequency data.

## Figures and Tables

**Table 1:** Summary of allelic comparisons from alignment of two phases of GB national data.

Locus	Allele Calls Compared	Alignment Factor (bp)	Allele Calls in Agreement with Alignment Factor (%)	Null Alleles (%)	Range of Error from Alignment Factor (bp)
CPPCT022	75	0	81%	13%	1
CPPCT006	84	0	99%	1%	n/a ^2^
EMPaS02	78	−3 or −4 ^1^	90%	8%	−1
BPPCT037	75	1	77%	3%	−1
EMPaS06	72	0	89%	4%	−1 to 1
EMPa004	83	0	90%	10%	n/a
EMPa017	68	0	82%	9%	1
EMPa018	72	0	94%	6%	n/a
EMPaS12	74	-8	95%	3%	1 or −7
EMPaS14	80	0	83%	16%	1

^1^ An allele-range-specific alignment factor was used for EMPaS02. ^2^ n/a: not applicable.

**Table 2:** Summary of allelic comparisons from alignment of national and central data.

Country	Allele Calls ^1^	EMPa002	CPSCT038	CPPCT022	CPPCT006	BPPCT034	EMPaS02	PAV-Rf-SSR	BPPCT037	EMPaS06	EMPaS12	EMPaS14	EMPa004	EMPa018	EMPa017
France	Compared	41	n/a	n/a	n/a	57	55	n/a	n/a	49	51	50	53	50	44
	In agreement (%)	90%	n/a	n/a	n/a	79%	95%	n/a	n/a	90%	78%	88%	94%	94%	89%
	Null (%)	10%	n/a	n/a	n/a	21%	5%	n/a	n/a	6%	18%	6%	6%	6%	7%
	**Total**	**100%**				**100%**	**100%**			**96%**	**96%**	**94%**	**100%**	**100%**	**95%**
Germany	Compared	72	n/a	78	82	n/a	73	n/a	83	79	77	76	n/a	n/a	57
	In agreement (%)	99%	n/a	97%	99%	n/a	100%	n/a	94%	95%	97%	99%	n/a	n/a	84%
	Null (%)	1%	n/a	0%	1%	n/a	0%	n/a	1%	0%	1%	0%	n/a	n/a	2%
	**Total**	**100%**		**97%**	**100%**		**100%**		**95%**	**95%**	**99%**	**99%**			**86%**
Great Britain	Compared	65	64	77	81	78	83	61	80	81	80	78	79	76	67
	In agreement (%)	80%	61%	86%	91%	74%	85%	46%	85%	96%	97%	95%	96%	97%	72%
	Null (%)	18%	36%	4%	3%	24%	5%	52%	3%	0%	1%	1%	4%	3%	6%
	**Total**	**98%**	**97%**	**89%**	**94%**	**97%**	**90%**	**98%**	**87%**	**96%**	**99%**	**96%**	**100%**	**100%**	**78%**
Italy	Compared	39	n/a	44	50	n/a	53	n/a	54	55	60	n/a	n/a	n/a	48
	In agreement (%)	97%	n/a	84%	78%	n/a	87%	n/a	69%	78%	82%	n/a	n/a	n/a	77%
	Null (%)	0%	n/a	5%	12%	n/a	6%	n/a	11%	5%	8%	n/a	n/a	n/a	2%
	**Total**	**97%**		**89%**	**90%**		**92%**		**80%**	**84%**	**90%**				**79%**
Sweden	Compared	41	n/a	34	39	n/a	n/a	n/a	43	n/a	39	n/a	n/a	n/a	n/a
	In agreement (%)	78%	n/a	91%	97%	n/a	n/a	n/a	65%	n/a	62%	n/a	n/a	n/a	n/a
	Null (%)	12%	n/a	9%	3%	n/a	n/a	n/a	16%	n/a	8%	n/a	n/a	n/a	n/a
	**Total**	**90%**		**100%**	**100%**				**81%**		**69%**				
Switzerland	Compared	n/a	n/a	15	12	n/a	15	n/a	14	14	14	13	n/a	n/a	n/a
	In agreement (%)	n/a	n/a	67%	83%	n/a	100%	n/a	93%	86%	93%	69%	n/a	n/a	n/a
	Null (%)	n/a	n/a	0%	0%	n/a	0%	n/a	7%	0%	0%	0%	n/a	n/a	n/a
	**Total**			**67%**	**83%**		**100%**		**100%**	**86%**	**93%**	**69%**			

^1^ Allele calls—’Compared’ indicates the total number of calls compared across all samples replicated between the national and central data (including calls marked in one or other dataset as null). n/a: not applicable (data not available).

**Table 3:** Summary statistics from allele frequency analysis of unique diploid samples after alignment.

Locus	Allele no.	N	H_O_	H_E_	PIC	NE-I	NE-SI
EMPa002	15	844	0.47	0.46	0.37	0.38	0.62
CPSCT038	6	209	0.52	0.54	0.49	0.26	0.54
CPPCT022	17	1111	0.66	0.68	0.62	0.16	0.45
CPPCT006	22	1122	0.73	0.75	0.70	0.11	0.40
BPPCT034	19	376	0.76	0.74	0.70	0.10	0.41
EMPaS02	17	1252	0.76	0.80	0.77	0.07	0.37
PAV-Rf-SSR	7	120	0.74	0.73	0.70	0.11	0.41
BPPCT037	22	1125	0.80	0.80	0.78	0.07	0.37
EMPaS06	16	1248	0.83	0.84	0.82	0.05	0.34
EMPaS12	17	1275	0.78	0.78	0.74	0.09	0.38
EMPaS14	13	1107	0.63	0.58	0.50	0.25	0.52
EMPa004	10	423	0.78	0.71	0.66	0.14	0.43
EMPa018	11	425	0.60	0.65	0.61	0.16	0.47
EMPa017	11	855	0.35	0.37	0.35	0.42	0.67
Mean	14.5	821	0.67	0.67	0.63	0.17	0.46

N: Number of individuals typed; H_O_: Observed homozygosity; H_E_: Expected heterozygosity; PIC: Polymorphic information content; NE-I: Average non-exclusion probability for identity of two unrelated individuals; NE-SI: Average non-exclusion probability for identity of two siblings.

**Table 4:** Total allele number from the whole dataset (including polyploid samples) after alignment.

Locus	Allele No.
EMPa002	30
CPSCT038	12
CPPCT022	29
CPPCT006	27
BPPCT034	32
EMPaS02	23
PAV-Rf-SSR	7
BPPCT037	30
EMPaS06	22
EMPaS12	28
EMPaS14	27
EMPa004	15
EMPa018	13
EMPa017	17

## Data Availability

All data used in the publication are available in the supplementary tables.

## Supplementary Materials

The following are available online at https://www.mdpi.com/article/10.3390/plants10061243/s1, Table S1: Alignment of the two phases of GB data, Table S2: Alignment of ‘EU.CHERRY’ and ‘Prunus Alignment’ replicates in the central dataset, Table S3: Combined dataset containing original and aligned scores from central and national datasets, Table S4: Alignment of replicates in national datasets to the central data, Table S5: Summary of final alignment factors and allele-specific adjustments across all alleles in national data, Table S6: Summary of Locus- or Allele-specific alignment factors.
